# Antitumor Effect of mTOR1/2 Dual Inhibitor AZD8055 in Canine Pulmonary Carcinoma

**DOI:** 10.3390/cancers17121991

**Published:** 2025-06-14

**Authors:** Tomokazu Nagashima, Kazuhiko Ochiai, Yuka Takizawa, Youta Koike, Takahiro Saito, Asumi Muramatsu, Daigo Azakami, Yukino Machida, Makoto Bonkobara, Toshiyuki Ishiwata, Masaki Michishita

**Affiliations:** 1 Department of Veterinary Pathology, Faculty of Veterinary Science, Nippon Veterinary and Life Science University, Tokyo 180-8602, Japan; to.nagashima0703nvlu@nvlu.ac.jp (T.N.); smhuirmaa-as@outlook.jp (A.M.); ymachida@nvlu.ac.jp (Y.M.); 2 Laboratory of Veterinary Hygiene, Faculty of Veterinary Science, Nippon Veterinary and Life Science University, Tokyo 180-8602, Japan; kochiai@nvlu.ac.jp; 3 Laboratory of Clinical Oncology, Tokyo University of Agriculture and Technology, Tokyo 183-8538, Japan; koitubomi58@gmail.com (Y.T.); koike014129@gmail.com (Y.K.); s258289q@st.go.tuat.ac.jp (T.S.); ft6225@go.tuat.ac.jp (D.A.); 4 Laboratory of Veterinary Clinical Pathology, Faculty of Veterinary Science, Nippon Veterinary and Life Science University, Tokyo 180-8602, Japan; bonkobara@nvlu.ac.jp; 5 Division of Aging and Carcinogenesis, Research Team for Geriatric Pathology, Tokyo Metropolitan Institute of Gerontology, Tokyo 173-0015, Japan; ishiwatatoshi@gmail.com; 6 One Health One Welfare Center, Nippon Veterinary and Life Science University, Tokyo 180-8602, Japan

**Keywords:** AZD8055, canine, lung, mTOR, PI3K/AKT/mTOR pathway, pulmonary adenocarcinoma

## Abstract

**Background/Objectives:** Primary pulmonary carcinoma (PC) is a malignant neoplasm that occurs in humans, dogs, and other species. In canine PC, palliative care remains the most practical approach for dogs with inoperable PC. **Methods:** We investigated the effectiveness of mammalian target of rapamycin (mTOR) inhibitors in canine lung cancer upon PI3K/AKT/mTOR activation. Three canine PC cell lines (AZACL1, AZACL2, and cPAC-1) were treated with three mTOR inhibitors (AZD8055, temsirolimus, and everolimus). In vitro, sensitivity assays were conducted to evaluate proliferation and Western blotting was used to examine pathway activation and phosphorylation of mTOR-related protein. **Results:** AZD8055 had a stronger inhibitory effect on cell proliferation than temsirolimus and everolimus in all three PC cell lines. The IC_50_ for AZD8055 in the AZACL1, AZACL2, and cPAC-1 cell lines were 23.8 μM, 95.8 nM, and 237 nM, for temsirolimus they were 34.6 μM, 11.5 μM, and 11.2 μM, and for everolims they were 36.6 μM, 33.4 μM, and 33.0 μM, respectively. Western blotting revealed PI3K/AKT/mTOR pathway activation and differential phosphorylation of mTOR signal-related proteins across the three PC cell lines. In xenograft mice injected with the AZACL1 and AZACL2 cell lines we showed that the AZD8055-treated group exhibited a significant reduction in tumor volume via the inhibition of tumor growth compared to the control group. **Conclusions:** These findings reveal that the PI3K/AKT/mTOR pathway plays a key role in canine PC and that AZD8055 may be a novel therapeutic agent for PC-bearing dogs.

## Simple Summary

Primary pulmonary carcinoma (PC) is a malignant neoplasm that occurs in humans, dogs, and other species. Palliative care in canine PC is the most practical approach for the dogs with inoperable PC. We investigated the effectiveness of mammalian target of rapamycin (mTOR) inhibitors in canine lung cancer upon PI3K/AKT/mTOR activation in three canine PC cell lines and three mTOR inhibitors (AZD8055, temsirolimus, and everolimus) in vitro and in vivo. This study revealed, for the first time, that the PI3K/AKT/mTOR pathway plays an important signaling pathway in canine PC and that AZD8055 may be a novel therapeutic agent for PC-bearing dogs. Further studies on mTOR inhibitors in canine PC may lead to personalized medicine for canine PC and serve as a useful model for research on human lung cancer.

## 1. Introduction

Primary pulmonary tumors account for 1% of all tumors in dogs, with approximately 85% classified as epithelial tumors [1], typically arising from the airway epithelium or alveolar parenchyma [2]. The therapeutic strategy for canine pulmonary carcinoma (PC) varies according to the anatomic location, clinical stage, and histologic type. Surgical resection is the most recommended therapy for primary pulmonary tumors in dogs and is currently the only curative-intent approach [3]. However, surgical excision is not always feasible or indicated because of its precise intrapulmonary location, invasion of neighboring organs, involvement of lymph nodes, or distant metastases at diagnosis [3]. Palliative care constitutes a viable treatment option for dogs with inoperable pulmonary cancer [1]. Despite several studies indicating that chemotherapy with vinorelbine [4] or metronomic therapy may be efficacious in canines with advanced pulmonary cancers, no medical treatment has been established as the gold standard [1].

Lung cancer is the leading cause of both incidence and mortality rates among individuals worldwide [5]. In humans, almost all lung cancers are carcinomas and histologically classified into small-cell lung cancers and non-small-cell lung cancers, the latter including adenocarcinoma, squamous-cell carcinoma, and large-cell neuroendocrine carcinoma as the main histologic subtypes [6]. Lung cancer therapies in humans have advanced rapidly over time, with a particular focus on precision medicine, leading to the classification of patient management based on biomarkers [7]. Currently, in lung adenocarcinoma predictive testing for driver mutations, which are mutations that spread cancer regardless of sex, race, smoking history, or other risk factors, is prioritized over other molecular predictive tests [6,8].

The phosphatidylinositol 3-kinase (PI3K), protein kinase B (PKB; AKT), and mammalian target of rapamycin (mTOR) signaling pathways mediate an array of fundamental cellular processes, such as proliferation, survival, nutrient uptake, metabolic activity, and anabolic reactions (protein synthesis) [9,10]. The PI3K/AKT/mTOR signal transduction pathway is constitutively active in many cancers, including breast, lung, and colon cancers [8,9]. Pathway aberrations are the most commonly activated pathway in human cancers, occurring in approximately 50% of cancers [8,9]. The two structurally and functionally distinct mTOR complexes are mTOR complex 1 (mTORC1) and mTOR complex 2 (mTORC2) [10]. mTORC1 is pivotal in cellular growth and metabolism, with its downstream signaling pathway promoting protein synthesis largely through the phosphorylation of two key effectors, p70S6 kinase 1 (S6K1) and eIF4E binding protein (4E-BP) [11]. The primary function of mTORC2 is likely the phosphorylation and activation of AKT, which promotes cell survival, proliferation, and growth. mTORC2 additionally has another downstream signaling pathway, which involves the phosphorylation of the PKC family [11]. Therefore, mTOR inhibitors are key candidates for human cancer therapy. Rapamycin analogs (rapalogs) have been approved for cancer treatment, and many mTOR inhibitors with different mechanisms of action, such as everolimus, temsirolimus, and AZD8055, have been developed [10,12]. Everolimus and temsirolims are developed first-generation mTOR inhibitors and are approved by the Food and Drug Administration to treat several human cancers, such as renal-cell carcinoma [9,10,12]. A second-generation mTOR inhibitor targeting mTORC1/2, AZD8055, has demonstrated antitumor activity against various advanced solid tumors in clinical studies [9,10,12]. However, to date, no studies have evaluated the efficacy of mTOR inhibitors in canine PC.

Similarly to human oncology, the increasing availability of molecular diagnostic tools for veterinary oncology is enhancing the comprehension of the role of PI3K/AKT/mTOR pathway activation in canine cancer [13]. In veterinary medicine, cancer therapy targeting the PI3K/AKT/mTOR signaling pathway is expected to exhibit antitumor effects against canine mammary cancer [14], hemangiosarcoma [15], and melanoma [16,17]. Although there have been few studies on the PI3K/AKT/mTOR pathway in canine PC, the results of this study may contribute to the establishment of novel therapies for canine PC and human lung cancer research.

The present study used three mTOR inhibitors, AZD8055, temsirolimus, and everolimus, to evaluate the in vitro and in vivo antitumor efficacies in canine PC cell lines. The mTORC1/2 dual-targeting inhibitor AZD8055 has demonstrated potential as a novel molecular-targeted inhibitor against canine PC.

## 2. Materials and Methods

### 2.1. Cell Lines and Culture

In this study, we used three canine PC cell lines. AZACL1 and AZACL2 were acquired from Cosmo Bio Co., Ltd. (Tokyo, Japan) and cPAC-1 was derived from an adenocarcinoma arising in the left caudal lung lobe of a 15-year-old neutered female mixed-breed dog. These cell lines were cultured in Dulbecco’s modified Eagle medium (FUJIFILM Wako Pure Chemical Corporation, Osaka, Japan), supplemented with 10% fetal bovine serum (Nichirei, Tokyo, Japan) and 1% antibiotics (Nakarai Tesque, Kyoto, Japan) at 37 °C in an atmosphere containing 5% CO_2_ environment.

### 2.2. In Vitro Sensitivity Assay of mTOR Inhibitors

AZACL1 and AZACL2 cell lines were seeded at 5 × 10^3^, while cPAC-1 cell lines at 1 × 10^4^ cells/well on 96-well plates. After 24 h of culture, cells were treated with a fresh culture medium containing six different doses (final concentration: 0.001, 0.01, 0.1, 1, 10, or 100 mM) of AZD8055 (Selleck Chemicals, Houston, TX, USA), temsirolimus (LC Laboratories, Woburn, MA, USA) or everolimus (LC Laboratories) for 48 h. Each living cell was evaluated using a Cell Counting Kit-8 (Dojindo Laboratories, Kumamoto, Japan). To analyze signaling activity following mTOR inhibitors treatment, AZACL1, AZACL2, and cPAC-1 cell lines were seeded at 1 × 10^5^, 1 × 10^5^, and 1 × 10^6^ in a 35 mm dish for culture, respectively. Adherent cultures were replaced with media containing each mTOR inhibitor at a final concentration of 10 μM after three days of culture. Cells were harvested 1 h and 4 h post-inhibitor addition, and Western blotting was performed as detailed below.

### 2.3. Western Blotting

Adherent cells derived from AZACL1, AZACL2, and cPAC-1 cells were collected by centrifugation and subsequently rinsed with phosphate-buffered saline. The adherent cells derived from AZACL1, AZACL2, and cPAC-1 cells were treated with or without mTOR inhibitors and lysed with mammalian lysis buffer (Promega Corporation, Madison, WI, USA) supplemented with a protease inhibitor cocktail (Roche, Basel, Switzerland) for 15 min. Protein concentrations were quantified using a bicinchoninic acid protein assay kit (Nacalai Tesque, Kyoto, Japan). The protein extract from the cells (5 μg) was mixed with 2× loading buffer and separated on 5–20% gradient sodium dodecyl sulfate-polyacrylamide gel electrophoresis (DRC, Tokyo, Japan). The isolated proteins were transferred onto polyvinylidene difluoride (PVDF) membranes. Membranes were blocked with BlockPRO blocking buffer (FUJIFILM Wako Pure Chemical Corporation) for 1 h at 25 °C. Western blotting was performed using the following primary antibodies for 16 h at 4 °C: rabbit monoclonal anti-Akt (Pan; 1:1000; cat. no. 4691, Cell Signaling Technology, Inc. Cambridge, MA, USA), phospho-Akt (Ser473; 1:1000; cat. no. 4060, CST), mTOR (1:1000; cat. no. 2983, CST), phospho-mTOR (Ser2448; 1:1000; cat. no. 2976, CST), 4E-BP (1:1000; cat. no. 9644, CST) and phospho-4E-BP (Thr37/46; 1:1000; cat. no. 2855, CST), rabbit monoclonal anti-TTF-1 (1:1000; cat. no. ab76013, abcam, Cambridge, UK), and mouse monoclonal anti-α-tubulin (1:2000; cat. no. 013-25033, FUJIFILM Wako Pure Chemical Corporation). The PVDF membrane was incubated with primary antibodies, followed by horseradish peroxidase-conjugated immunoglobulin G (IgG) (GE Healthcare, Tokyo, Japan) for 1 h at 25 °C. Antibody-bound proteins were detected using EzWestLumi Plus (ATTO, Tokyo, Japan), and chemiluminescence images were captured using Vilber Bio-Imaging FUSION (Collégien, France).

### 2.4. Oral Administration of AZD8055 to the Tumor Xenograft Model

Eight-week-old female BALB/c nude mice were purchased from CLEA Inc. (Tokyo, Japan). A suspension of 1 × 10^6^ adherent cells derived from AZACL1, AZACL2, and cPAC-1 was subcutaneously injected into the ventrolateral area under anesthesia. AZD8055 was dissolved in 30% (*w*/*v*) sulfobutylether-β-Cyclodextrin (SBE-β-CD, Angene International Limited, Nanjing, China) and administered orally to mice at a dose of 20 mg/kg per day for 31 days following macroscopic confirmation of tumor formation (*n* = 6). The in vivo dose of AZD8055 was determined based on the previous studies [18]. The control group (*n* = 6) received the vehicle only. For each of the three PC cell lines, mice were divided into control and AZD8055-treated groups, with a total of 36 animals used in this study. Tumor volume (V) was estimated using the following equation: V = [(length) × (width)^2^]/2. The in vivo antitumor effects of mTOR inhibitors were evaluated in two cell lines, AZACL1 and AZACL2, capable of tumor formation via xenograft transplantation. The experiments were approved by the Animal Experiments Committee of Nippon Veterinary and Life Science University and were performed in compliance with the Guidelines for Animal Experiments of the Nippon Veterinary and Life Science University.

### 2.5. Histopathology

The tumors developed in nude mice administered with AZACL1 and AZACL2 cell lines were fixed with 10% neutral-buffered formalin and routinely embedded in paraffin wax for histological examination. The sections were stained with hematoxylin and eosin. Serial sections were immunostained using the streptavidin–biotin–peroxidase method with primary monoclonal antibodies specific for Ki-67 (1:100; Dako, Denmark A/S, Glostrup, Denmark). The sections were treated with 0.3% H_2_O_2_ in 33% methanol at room temperature for 30 min to block endogenous peroxidase activity, followed by antigen retrieval at 121 °C for 20 min in citrate buffer (pH 6.0). The validation of antibodies was confirmed by a positive reaction with biopsy samples diagnosed with canine pulmonary adenocarcinoma or by a negative normal mouse IgG. The Ki-67 labeling index was evaluated in five hotspots (high-power field) and defined as the ratio of positive tumor cells to the total number of tumor cells using the ImageJ 1.54p software.

### 2.6. Statistical Analysis

The results are presented as means ± standard deviation. The Kolmogorov–Smirnov test was used to test for normal distribution, while the *F* test was used to detect equal distribution. Statistical analyses were conducted using Student’s *t*-test and Welch’s *t*-test in R version 4.5.0. A value of *p* < 0.05 was deemed indicative of a statistically significant difference.

## 3. Results

### 3.1. Detection of TTF-1 Expression of Canine PC Cell Lines

**Figure 1:**
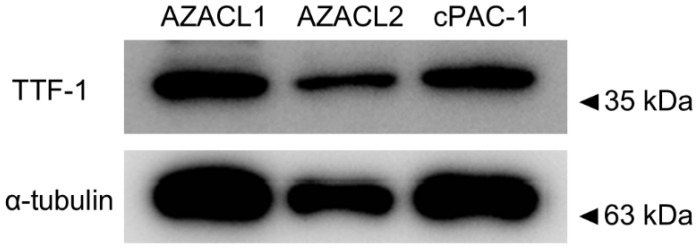
Western blot analysis of TTF-1 in canine pulmonary carcinoma cell lines, AZACL1, AZACL2, and cPAC-1.

Western blotting was performed to confirm the expression of thyroid transcription factor (TTF-1), a marker of primary lung tumors. TTF-1 expression was detected as a single band in all canine PC cell lines (Figure 1).

### 3.2. In Vitro Antitumor Effects of mTOR Inhibitors on the Proliferation of Canine PC Cell Lines

**Figure 2:**
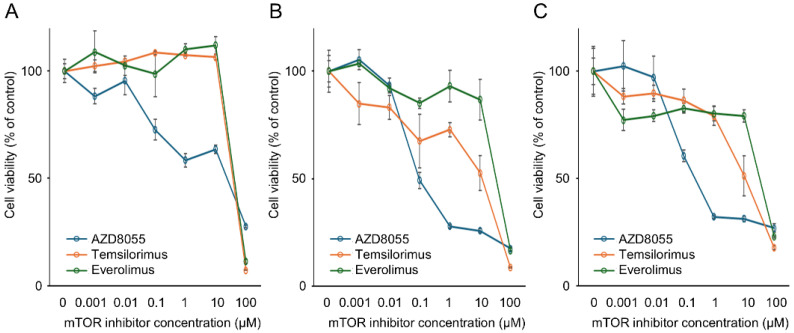
In vitro sensitivity assay of mTOR inhibitors, including everolimus, temsirolimus, and AZD8055, in canine pulmonary carcinoma cell lines: (**A**) AZACL1, (**B**) AZACL2, and (**C**) cPAC-1. The results are representative of at least three independent experiments. Data are calculated as the mean ± SD.

A sensitivity assay was performed in vitro with the mTOR inhibitors AZD8055, temsirolimus, and everolimus to examine their inhibitory effects on PC cell line proliferation. The AZACL1, AZACL2, and cPAC-1 cell lines exhibited a dose-dependent decrease in cell viability following treatment with AZD8055, temsirolimus, and everolimus (Figure 2). The IC_50_ values for AZD8055 in the AZACL1, AZACL2, and cPAC-1 cell lines were 23.8 μM, 95.8 nM, and 237 nM, respectively. For temsirolimus, the IC_50_ values were 34.6 μM, 11.5 μM, and 11.2 μM, and those of everolimus were 36.6 μM, 33.4 μM, and 33.0 μM, respectively. The IC_50_ of AZD8055 was lower than that of temsirolimus and everolimus in the three cell lines.

### 3.3. mTOR1/2 Inhibition AZD8055 Strongly Suppresses Phosphorylation of mTOR Signal Compared with mTOR1 Inhibitors Everolimus and Temsirolimus

**Figure 3:**
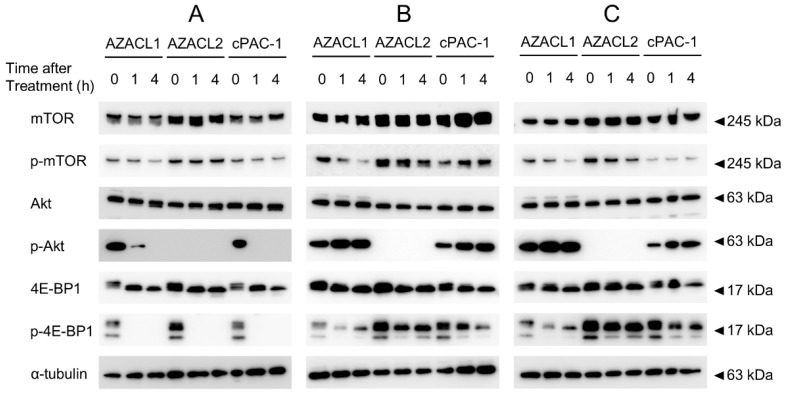
Western blot analysis of mTOR signaling pathway proteins following treatment with (**A**) AZD8055, (**B**) temsirolimus, and (**C**) everolimus in the canine pulmonary carcinoma cell lines AZACL1, AZACL2, and cPAC-1. Expression levels of mTOR, Akt, and 4E-BP1 showed similarity in all three cell lines. Their phosphorylated proteins are expressed in almost all PC cell lines, except for phosphorylated Akt in AZACL2. Compared with everolimus and temsirolimus, AZD8055 markedly reduced the phosphorylation of mTOR signaling-related proteins in all of the cell lines.

Western blotting was performed to confirm the expression of mTOR signaling-related proteins and their phosphorylated counterparts following mTOR inhibitor treatment in PC cell lines (Figure 3). The expression levels of mTOR, Akt, and 4E-BP1 proteins were similar among the three PC cell lines. Furthermore, their phosphorylated proteins were confirmed to be expressed in almost all PC cell lines, except for phosphorylated Akt in the AZACL2 cell line. Upon AZD8055 treatment, a time-dependent decrease in both phosphorylated Akt and 4E-BP1 was observed in the AZACL1 and cPAC-1 cell lines. In AZACL2 cells, only phosphorylated 4E-BP1 showed a time-dependent reduction in expression following AZD8055 treatment. Treatment with everolimus and temsirolimus resulted in a slight decrease in phosphorylated mTOR and 4E-BP1 in the AZACL1 cell line compared to untreated controls; however, no significant decreases in the phosphorylation of mTOR and its downstream signals were observed in the AZACL2 and cPAC-1 cell lines.

### 3.4. In Vivo Antitumor Effects of AZD8055 in Xenograft Mice Injected with PC Cell Lines

**Figure 4:**
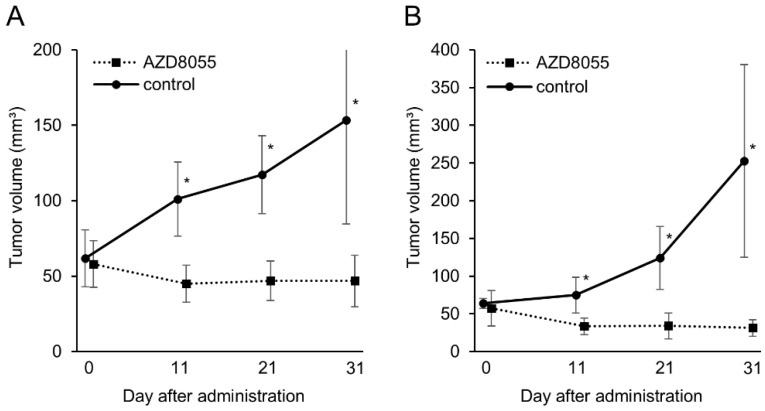
Antitumor effects of AZD8055 in a xenograft model of the transplanted canine pulmonary carcinoma cell lines (**A**) AZACL1 and (**B**) AZACL2. AZD8055 (*n* = 6, squares) or control (*n* = 6, circles) was administrated for 31 days. The differences were tested by Student *t*-test and Welch’s *t*-test. * *p* < 0.05 indicates statistical significance.

To elucidate the antitumor effect of AZD8055 in vivo, AZD8055 was administered to xenografted mice injected with the AZACL1 and AZACL2 cell lines. In the AZACL1 cell line, the tumor volumes in the control and AZD8055-treated groups at the end of treatment were 153 ± 68.7 mm^3^ and 46.8 ± 16.9 mm^3^, respectively (Figure 3). In the AZACL2 cell line, the tumor volumes in the control and AZ8055-treated groups at the end of treatment were 253 ± 127 mm^3^ and 31 ± 10.9 mm^3^, respectively (Figure 4). In both the AZACL1 and AZACL2 cell lines, the AZ8055-treated cohort exhibited a significant reduction in tumor volume compared with the control group. Tumor formation was not observed in mice injected with the cPAC-1 cell line, and therefore, the in vivo antitumor effect of AZD8055 could not be evaluated.

**Figure 5:**
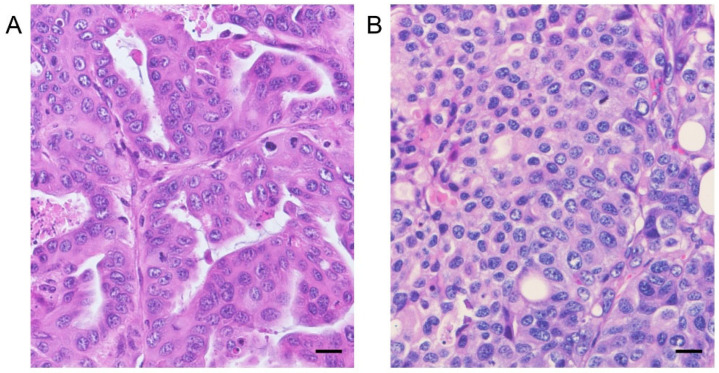
Histopathological evaluation of xenograft tumor formed by canine lung cancer pulmonary carcinoma cell lines in mice. Tumors formed in the xenografts mice injected with the AZACL1 cell line consisted of glandular cells (**A**), while in mice injected with the AZACL2 cell line they consisted of cells showing a solid pattern (**B**). Hematoxylin and eosin. Scale bar = 20 μm.

**Figure 6:**
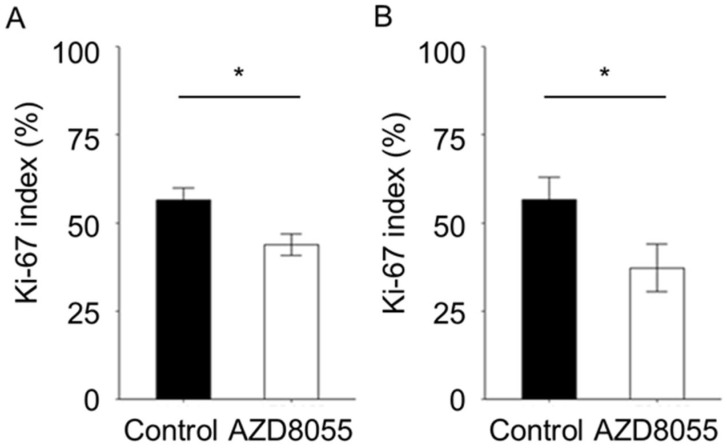
Immunohistochemical analysis of Ki-67 expression in xenograft tissue from the (**A**) AZACL1 and (**B**) AZACL2 cell lines following oral administration of AZD8055. Comparison of the Ki-67 labeling index calculated as the ratio of positive cells to total cells in the AZACL1 and AZACL2 cell lines xenografted tissues of the vehicle and 20 mg/kg AZD8055 treated-groups. For comparison between the groups, Student *t*-test and Welch’s *t*-test were performed. All data were calculated as the mean ± SD. * indicates a significant difference between two groups: * *p* < 0.05.

The tumors formed in mice injected with the AZACL1 cell line demonstrated glandular-to-nested tumor cell proliferation, while those in mice injected with the AZACL2 cell line demonstrated solid tumor cell proliferation (Figure 5). In the AZACL1 and AZACL2 cell lines, tumor necrosis and infiltration of inflammatory cells, including lymphocytes and mast cells, were observed to a comparable degree in both the control and AZD8055-treated groups. The Ki-67 indices of tumor cells in the control and AZD8055-treated groups were 56.5 ± 3.43 and 43.9 ± 3.07 for the AZACL1 cell line, and 56.6 ± 6.38 and 45.4 ± 6.74 for AZACL2 cell line, respectively (Figure 6). The Ki-67 index was significantly different between the control and AZD8055-treated groups in both the AZACL1 and AZACL2 cell lines (*p* < 0.05).

## 4. Discussion

In this study, we investigated the antitumor efficacy of three mTOR inhibitors across three PC cell lines. Everolimus and temsirolimus are first-generation allosteric (noncompetitive) mTOR inhibitors, while AZD8055 is a second-generation ATP-competitive mTOR inhibitor [9]. Allosteric inhibitors only target mTORC1, rendering them incapable of circumventing the feedback loop-based induction of AKT, which is determined by the suppression of mTORC1 [9,19]. Moreover, allosteric mTOR inhibitors can modestly reduce phosphorylated 4E-BP1 levels by inhibiting its phosphorylation and, as a result, are unable to effectively inhibit eIF4E-mediated cap-dependent translation initiation in human cancers, including head and neck squamous-cell carcinomas [9,20]. ATP-competitive mTOR inhibitors can target both mTORC1 and mTORC2, avoiding the feedback loop-based induction of AKT, as determined by the suppression of mTORC1 [9,19]. ATP-competitive mTOR inhibitors markedly reduce phosphorylated 4E-BP1 and can effectively prevent eIF4E-mediated cap-dependent translation initiation in cancer. Furthermore, an ATP-competitive mTOR inhibitor promotes the active dephosphorylation of Thr46 of 4E-BP1, re-establishing its endogenous functions of growth inhibition and pro-apoptosis [9,20]. Therefore, these inhibitors elicit more potent suppression of PI3K/AKT/mTOR signaling compared to allosteric mTOR inhibitors, leading to enhanced antitumor activity [9,19]. Several clinical trials have been conducted or are underway to evaluate the therapeutic efficacy of mTOR inhibitors, including everolimus and temsirolimus, in human cancers [9,17]. This study demonstrated that AZD8055 treatment revealed a stronger inhibitory effect on cell proliferation compared everolimus and temsirolimus while also reducing the expression of phosphorylated 4E-BP1 in all three lines in vitro. In a previous study, AZD8055 significantly reduced phosphorylated 4E-BP1 levels in various human tumor cell lines [18]. Rapamycin, another mTOR inhibitor, causes sustained inhibition of S6K phosphorylation but has a limited effect on sensitive phosphorylation sites (T37/46) of 4E-BP1 and may even lead to over-phosphorylation of 4E-BP1 over time following its administration [21]. The variations in the antitumor efficacy of mTOR inhibitors in this study may be due to differences in the inhibition levels of phosphorylated 4E-BP1 among mTOR inhibitors. Furthermore, AZD8055 potently inhibits AKT phosphorylation via inhibition of mTORC2 [18], suggesting that AZD8055 strongly attenuated phosphorylated AKT in the AZACL1 and cPAC-1 cell lines in the present study. AZD8055 demonstrated a strong antitumor effect against the AZACL2 cell line, which lacks AKT phosphorylation. The PI3K/AKT pathway is an important activator of mTOR; nevertheless, multiple signaling pathways regulate mTOR independent of AKT [22]. The antitumor effect of AZD8055 against AZACL2 may have been due to the inhibition of AKT-independent pathways.

In addition to the in vitro and Western blot analyses, we demonstrated the antitumor effects of AZD8055 in PC-transplanted mice, where AZD8055-treated groups showed a significant reduction in tumor volume via the inhibition of tumor growth compared to the control group in xenograft mice transplanted with the AZACL1 and AZACL2 cell lines. The in vivo antitumor effect of AZD8055 against xenograft mice injected with the cPAC-1 cell line could not be evaluated in this study because tumor formation was not observed in the xenograft. The poor engraftment of cPAC-1 may be attributed to its dependence on specific extracellular matrix components, growth factors, or cytokines, or to inherent properties that reduce its capacity to survive in vivo. No obvious side effects were observed in the mice treated with AZD8055. While mTOR inhibitors exhibit promising efficacy in human preclinical studies, some inhibitors have serious side effects and must be discontinued [12]. Most side effects associated with mTOR inhibitors are mild or moderate, dose-dependent, and many are reversible upon the cessation of treatment [23]. The side effects of mTOR inhibitors in canines remain unknown and require further investigation. This study suggests that the PI3K/AKT/mTOR pathway is active in canine PC, and that AZD8055 may be a novel therapeutic agent for PC-bearing dogs. In addition, mTOR inhibitors in canine PC may serve as an animal model for human lung cancer research.

## 5. Conclusions

In conclusion, this study reveals, for the first time, the antitumor effects of mTOR inhibitors on PC cell lines both in vitro and in vivo. The in vitro analyses indicated variability in the phosphorylation of mTOR signal-related proteins across PC cell lines, with AZD8055 demonstrating stronger antitumor effects compared to temsirolimus and everolimus. Furthermore, in vivo experiments revealed that AZD8055 induced tumor regression in PC-xenografted mice. A detailed analysis of the mTOR signaling pathway-related proteins and the antitumor effect of AZD8055 may lead to the development of personalized medicine for canine PC.

## Data Availability

The data supporting the findings of this study are available from the corresponding author upon reasonable request.

## Abbreviations

Abbreviation	Full Form
PC	Pulmonary carcinoma
PI3K	Phosphatidylinositol 3-kinase
PKB; AKT	Protein kinase B
mTOR	Mammalian target of rapamycin
mTORC1	mTOR complex 1
mTORC2	mTOR complex 2
TTF-1	Thyroid transcription factor 1

## References

[1] Rinaldi V., Finotello R., Boari A., Cabibbo E., Crisi P.E. (2023). Vinorelbine as first-line treatment in stage IV canine primary pulmonary carcinoma. Vet. Sci..

[2] Meuten D.J. (2017). Tumors in Domestic Animals.

[3] Polton G., Finotello R., Sabattini S., Rossi F., Laganga P., Vasconi M.E., Barbanera A., Stiborova K., Rohrer Bley C., Marconato L. (2018). Survival analysis of dogs with advanced primary lung carcinoma treated by metronomic cyclophosphamide, piroxicam, and thalidomide. Vet. Comp. Oncol..

[4] Wouda R.M., Miller M.E., Chon E., Stein T.J. (2015). Clinical effects of vinorelbine administration in the management of various malignant tumor types in dogs: 58 cases (1997–2012). J. Am. Vet. Med. Assoc..

[5] Bray F., Laversanne M., Sung H., Ferlay J., Siegel R.L., Soerjomataram I., Jemal A. (2024). Global Cancer Statistics 2022: GLOBOCAN estimates of incidence and mortality worldwide for 36 cancers in 185 countries. CA Cancer J. Clin..

[6] Tang M., Abbas H.A., Negrao M.V., Ramineni M., Hu X., Hubert S.M., Fujimoto J., Reuben A., Varghese S., Zhang J. (2021). The histologic phenotype of lung cancers is associated with transcriptomic features rather than genomic characteristics. Nat. Commun..

[7] WHO Classification of Tumours Editorial Board (2021). Thoracic Tumours.

[8] Martini M., De Santis M.C., Braccini L., Gulluni F., Hirsch E. (2014). PI3K/AKT signaling pathway and cancer: An updated review. Ann. Med..

[9] Glaviano A., Foo A.S.C., Lam H.Y., Yap K.C.H., Jacot W., Jones R.H., Eng H., Nair M.G., Makvandi P., Geoerger B. (2023). PI3K/AKT/mTOR signaling transduction pathway and targeted therapies in cancer. Mol. Cancer.

[10] Zou Z., Tao T., Li H., Zhu X. (2020). mTOR signaling pathway and mTOR inhibitors in cancer: Progress and challenges. Cell Biosci..

[11] Saxton R.A., Sabatini D.M. (2017). mTOR signaling in growth, metabolism, and disease. Cell.

[12] Hua H., Kong Q., Zhang H., Wang J., Luo T., Jiang Y. (2019). Targeting mTOR for cancer therapy. J. Hematol. Oncol..

[13] Meuten T.K., Dean G.A., Thamm D.H. (2024). Review: The PI3K-AKT-mTOR signal transduction pathway in canine cancer. Vet. Pathol..

[14] Michishita M., Ochiai K., Nakahira R., Azakami D., Machida Y., Nagashima T., Nakagawa T., Ishiwata T. (2023). mTOR Pathway as a potential therapeutic target for cancer stem cells in canine mammary carcinoma. Front. Oncol..

[15] Maeda M., Ochiai K., Michishita M., Morimatsu M., Sakai H., Kinoshita N., Sakaue M., Onozawa E., Azakami D., Yamamoto M. (2022). In vitro anticancer effects of alpelisib against PIK3CA-mutated canine hemangiosarcoma cell lines. Oncol. Rep..

[16] Wei B.R., Michael H.T., Halsey C.H., Peer C.J., Adhikari A., Dwyer J.E., Hoover S.B., El Meskini R., Kozlov S., Weaver Ohler Z. (2016). Synergistic targeted inhibition of MEK and dual PI3K/mTOR diminishes viability and inhibits tumor growth of canine melanoma underscoring its utility as a preclinical model for human mucosal melanoma. Pigment Cell Melanoma Res..

[17] Bernard S., Poon A.C., Tam P.M., Mutsaers A.J. (2021). Investigation of the effects of mTOR inhibitors rapamycin and everolimus in combination with carboplatin on canine malignant melanoma cells. BMC Vet. Res..

[18] Chresta C.M., Davies B.R., Hickson I., Harding T., Cosulich S., Critchlow S.E., Vincent J.P., Ellston R., Jones D., Sini P. (2010). AZD8055 Is a potent, selective, and orally bioavailable ATP-competitive mammalian target of rapamycin kinase inhibitor with In vitro and In vivo antitumor activity. Cancer Res..

[19] Hsu P.P., Kang S.A., Rameseder J., Zhang Y., Ottina K.A., Lim D., Peterson T.R., Choi Y., Gray N.S., Yaffe M.B. (2011). The mTOR-regulated phosphoproteome reveals a mechanism of mTORC1-mediated inhibition of growth factor signaling. Science.

[20] Wang Z., Feng X., Molinolo A.A., Martin D., Vitale-Cross L., Nohata N., Ando M., Wahba A., Amornphimoltham P., Wu X. (2019). 4E-BP1 is a tumor suppressor protein reactivated by mTOR inhibition in head and neck cancer. Cancer Res..

[21] Choo A.Y., Yoon S.O., Kim S.G., Roux P.P., Blenis J. (2008). Rapamycin differentially inhibits S6Ks and 4E-BP1 to mediate cell-type-specific repression of mRNA translation. Proc. Natl. Acad. Sci. USA.

[22] Memmott R.M., Dennis P.A. (2009). Akt-dependent and -independent mechanisms of mTOR Regulation in cancer. Cell. Signal..

[23] Kaplan B., Qazi Y., Wellen J.R. (2014). Strategies for the management of adverse events associated with mTOR inhibitors. Transplant. Rev..

